# Evaluation of Intraocular Lens Tilt and Decentration in Congenital Ectopia Lentis by the Pentacam Scheimpflug System

**DOI:** 10.1155/2022/7246730

**Published:** 2022-03-11

**Authors:** Huiwen Ye, Zhenzhen Liu, Qianzhong Cao, Zhangkai Lian, Xinyu Zhang, Danying Zheng, Guangming Jin

**Affiliations:** State Key Laboratory of Ophthalmology, Zhongshan Ophthalmic Center, Sun Yat-sen University, Guangdong Provincial Key Laboratory of Ophthalmology and Visual Science, Guangdong Provincial Clinical Research Center for Ocular Disease, Guangzhou 510060, China

## Abstract

**Purpose:**

The purpose of this study was to quantify the characteristics of the tilt and decentration of the IOL after trans-scleral suture fixation surgery in congenital ectopia lentis (CEL) patients.

**Methods:**

The clinical characteristics of 70 CEL patients at Zhongshan Ophthalmic Center in China were retrospectively analyzed. The tilt and decentration of intraocular lens (IOL) were measured by using a Pentacam and compared between different axial length (AL) subgroups. The correlation between IOL tilt, decentration, and ocular characteristics was investigated using Spearman's correlation analysis.

**Results:**

The postoperative IOL position of CEL patients was mainly located nasally inferiorly. The average tilt of the IOL in CEL patients was less than 7° (for temporal: 2.21 ± 1.53°, for nasal: −1.84 ± 2.04°, for superior: 2.22 ± 2.18°, and for inferior: −1.70 ± 1.62°), and the average decentration of the IOL in CEL patients was larger than 0.4 mm (for temporal: 0.49 ± 0.38 mm, for nasal: −0.69 ± 0.46 mm, for superior: 0.72 ± 0.58 mm, and for inferior: −0.68 ± 0.54 mm). The decentration of CEL patients in the AL ≥ 26 subgroup was greater than those with AL < 24 mm and AL 24 to 26 mm subgroups (for superior: 0.72 ± 0.28 mm vs. 0.46 ± 0.25 mm and 0.48 ± 0.22 mm, all *P* < 0.05; for inferior: -0.94 ± 0.56 mm vs. −0.44 ± 0.26 mm and -0.44 ± 0.46 mm, all *P* < 0.05). IOL decentration was positively correlated with AL (for superior: *r* = 0.44, *P*=0.019; for inferior: *r* = 0.54, *P*=0.006). IOL tilt was positively correlated with AL on the superior side (*r* = 0.38, *P*=0.041).

**Conclusions:**

The extent of IOL decentration after trans-scleral suture fixation was great in CEL patients, and the IOL decentration in CEL patients was significantly associated with AL.

## 1. Introduction

Congenital ectopia lentis (CEL) is a rare disease that is defined as the dislocation or displacement of the natural crystalline lens [1]. CEL can occur as an ocular symptom of many systemic or hereditary diseases such as homocystinuria, Weill–Marchesani syndrome, and sulfite oxidase deficiency [2–4] with Marfan syndrome being the most common etiology [1, 5, 6].

Scleral suture-fixed posterior chamber intraocular lens (IOL) surgery is one of the most commonly performed surgical procedures for CEL patients. Although most patients can achieve good visual outcomes with this surgical technology, more complications have been reported compared to the traditional surgery method, in which the IOL is implanted into the capsular bag, and the position of the IOL is one of the most important complications affecting visual quality [7, 8]. As previous studies have shown, IOL decentration greater than 0.4 mm and tilt greater than 7° could induce a lower visual performance [9]. Korynta found that an IOL tilt of 12° and decentration of 3 mm could result in a postoperative myopia of −7.0D and astigmatism of +4.0D [10]. Hence, the position of the IOL is important for a good visual prognosis.

However, few studies have focused on the characteristics and factors associated with IOL tilt and decentration in CEL patients. Hence, this study was conducted to evaluate the characteristics of IOL tilt and decentration three months postoperatively and to investigate the factors associated with IOL tilt and decentration in CEL patients.

## 2. Methods

### 2.1. Design

This retrospective study was conducted at the Zhongshan Ophthalmic Center. All participants had signed an informed consent form. The study was approved by the Human Research Ethics Committee of the Zhongshan Ophthalmic Center and followed the principles of the Declaration of Helsinki.

### 2.2. Patients

The data from baseline and routine follow-up at three months postoperatively were obtained by reviewing the medical records of CEL patients who underwent scleral suture-fixed posterior chamber intraocular lens surgery from April 2019 to March 2021, and for those who underwent binocular surgery, the first eye at three months postoperatively was selected in this study. The included CEL patients were stratified into three categories according to AL: AL < 24 mm group, 24<AL < 26 mm group, and AL ≥ 26 mm group. The inclusion criteria were as follows: (1) patients diagnosed with bilateral EL; (2) patients younger than 40 years old; (3) patients who underwent trans-scleral suture fixation surgery; and (4) patients with adequately and asymmetrically dilated pupils during the Pentacam examination. The exclusion criteria were as follows: (1) patients with secondary lens dislocations or lens dislocations caused by ocular trauma; (2) patients with ocular surgery history; (3) patients with ocular trauma history; (4) patients who cannot cooperate in the examination; and (5) patients with unclear Pentacam images. The study was approved by the institutional review board of Zhongshan Ophthalmic Center and conducted in accordance with the Declaration of Helsinki.

### 2.3. Study Procedures

During the three months of postoperative routine follow-up, nonmydriatic and mydriatic photographs of the anterior segment were collected. AL and anterior chamber depth (ACD) were measured using an IOLMaster 700 (Carl Zeiss Meditec AG). The tilt and decentration of the IOLs were measured using the Pentacam HR system (Oculus Inc., Wetzlar, Germany). The Pentacam exam requires the patient to sit in front of the instrument with the pupil sufficiently dilated to obtain scanned slit images in 25 directions to obtain an image of the anterior and posterior segments of the lens. Image processing software (MATLAB) was used to fit the anterior and posterior surfaces of the IOL and the iris plane in each image, and the corresponding tilt and decentration were then calculated according to previously reported methods [11, 12]. The midpoint of the line connecting the two ends of the iris segment is considered as the pupil center, and the line passing through the pupil center and the anterior corneal center of curvature is defined as the pupillary axis. The perpendicular line at the midpoint of the intersection of the fitted lines on the anterior and posterior surfaces of the IOL is defined as the IOL axis. The IOL tilt is defined as the angle between the IOL axis and the pupillary axis around the *x*-axis or *y*-axis, while the decentration of the IOL is defined as the vertical distance from the IOL axis to the pupillary axis [13]. The vertical meridian is defined as the 90-degree meridian, and the horizontal meridian is defined as the 180-degree meridian. Positive values indicate the temporal side in the horizontal meridian and the superior side in the vertical meridian, while negative values indicate the nasal and inferior sides, respectively [14].

### 2.4. Surgical Technique

All patients underwent trans-scleral suture-fixed posterior chamber intraocular lens IOL implantation right after the crystalline lens extraction by a single experienced surgeon (DY Z). The fixation of the IOL haptic occurred using an 8–0 polypropylene suture (Johnson and Johnson, USA) with a 26-G needle, and the needles were pierced from the ciliary sulcus to the sclera of the 150-degree meridian and the 330-degree meridian from 2 mm posterior to the surgical limbus. All patients were implanted with 970 C/920H (Rayner, UK) one-piece IOLs. The bilateral sutures were evenly tightened so that the IOL was centered as much as possible. The end of the suture under the scleral flap was knotted in turn to bury the knot under the sclera. The scleral flap was closed with a 10/0 nylon suture.

### 2.5. Statistical Analysis

Statistical analysis of the quantitative data was performed for all variables, and the differences in ocular data between the three subgroups were tested by one-way Anova analysis. A Spearman correlation analysis was used to evaluate the correlation between the positions of the IOL and AL. All data were analyzed by using SPSS 23.0 (SPSS, IBM Corp, Armonk, NY, USA). The *P* value less than 0.05 was considered statistically significant.

## 3. Results

### 3.1. Demographic and Ocular Characteristics

The demographic data and ocular biometric parameters of the patients are shown in Table 1. A total of 70 eyes of 70 patients were enrolled. The mean age of CEL patients was 9.77 years ±5.13 (SD), and 88.57% (62/70) of patients were over 6 years. The mean AL was 24.86 ± 1.80 mm, and the mean ACD was 4.67 ± 1.45 mm, and six patients (8.57%) had undergone anterior segment vitrectomy during surgery.

### 3.2. IOL Tilt and Decentration

**Figure 1:**
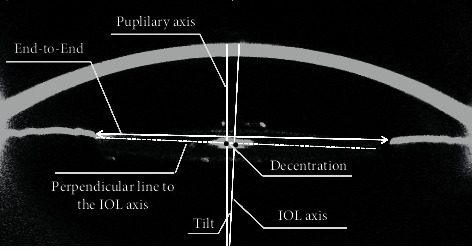
Fitted circumferences, pupillary axis, and IOL axis merged on a Scheimpflug cross-section image. Decentration is calculated from the distance between the IOL axis and pupillary axis. The IOL tilt was calculated from the angle between the axes.

**Figure 2:**
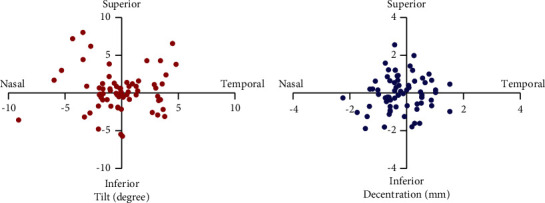
Degree and distribution of IOL tilt and decentration in CEL patients.

**Figure 3:**
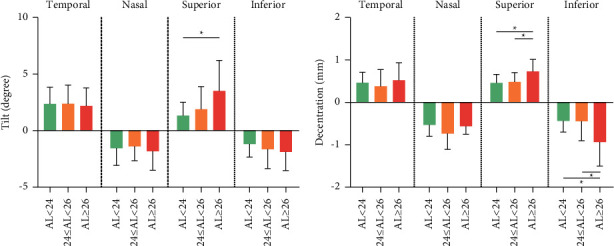
Comparisons of IOL tilt and decentration according to axial length in CEL patients.

**Figure 4:**
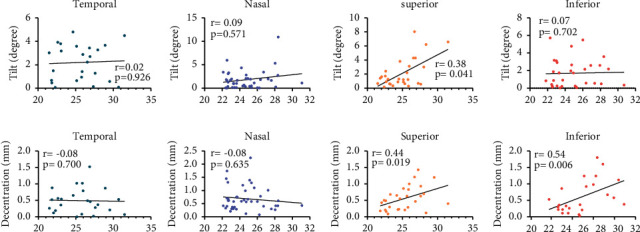
The scatter plots show the correlations between IOL position and AL at different directions.

Figure 1 illustrates the definition of IOL tilt and decentration. Tilt and decentration represent the angle and distance around the *x*-axis or *y*-axis of the IOL axis from the pupillary axis. Positive values indicate tilt and decentration exist on the nasal or temporal side, while negative values indicate tilt and decentration exist on the superior side or inferior side.  Figure 2 shows that the IOL tilt and decentration of CEL patients were mainly distributed nasally inferiorly (for tilt and decentration: both 23/70 (32.86%)). The average values of the IOL tilt and decentration in the CEL patients are shown in Table 2 and Figure 3. The mean tilt of the IOL in CEL patients was less than 7° (for temporal: 2.21 ± 1.53°, for nasal: −1.84 ± 2.04°, for superior: 2.22 ± 2.18°, and for inferior: −1.70 ± 1.62°), and the average decentration of the IOL in CEL patients was larger than 0.4 mm (for temporal: 0.49 ± 0.38 mm, for nasal: −0.69 ± 0.46 mm, for superior: 0.72 ± 0.58 mm, and for inferior: −0.68 ± 0.54 mm). The decentration in the AL ≥ 26 subgroup was greater than those with AL < 24 mm and 24<AL < 26 mm subgroups. (for superior: 0.72 ± 0.28 mm vs. 0.46 ± 0.25 mm and 0.48 ± 0.22 mm, all *P* < 0.05; for inferior: −0.94 ± 0.56 mm vs. −0.44 ± 0.26 mm and -0.44 ± 0.46 mm, all *P* < 0.05). The results of the correlations between the IOL position and the ocular meters are shown in Table 3 and Figure 4. AL was positively associated with IOL decentration at the superior and inferior side (for superiorly: *r* = 0.44, *P*=0.019; for inferiorly: *r* = 0.54, *P*=0.006), and AL was positively associated with IOL tilt at the superior side (*r* = 0.38, *P*=0.041).

## 4. Discussion

In recent years, Pentacam has been commonly used in clinical practice to measure IOL tilt and decentration. By using the Scheimpflug camera scanning principle, Pentacam can obtain a three-dimensional image of the anterior segment of the eye in less than two seconds, as well as automatically track and correct the patient's eye movements during the examination, and its measurements have been popularly commercialized [15]. In this study, by using Pentacam, we found that the postoperative IOL positions in CEL patients were mainly located nasally inferiorly, while the average tilt of IOLs in CEL patients was less than 7° in all four directions. However, the IOL decentration in CEL patients was larger than 0.4 mm. Moreover, patients with the longer AL had a greater IOL decentration than those with the shorter AL.

Previous studies have suggested that although small tilt and decentration of the IOL will not lead to abnormal visual experiences, large tilt and decentration can cause higher-order aberrations and changes in refractive status, which can affect the visual quality of patients [16–18]. Hence, maintaining a central position of the IOL in the visual axis region is particularly critical for postoperative visual prognosis. Although previous studies have suggested that the AL, ACD, and IOL types are related to IOL tilt and decentration, to our knowledge, studies on the distribution of IOL tilt and decentration in CEL patients after IOL scleral suture loop fixation and related factors are still rare.

Hayashi K's study showed that the tilt and decentration of the IOL after out-of-the-bag implantation were significantly greater than those after in-the-bag implantation, and the extent of both tilt and decentration after scleral suture fixation was greater than that observed after either out-of-the-bag or in-the-bag implantation [19, 20], which was consistent with part of our findings. In comparison with data in the published literature, our study showed that the tilt of the IOL after trans-scleral fixation in CEL patients was not significantly greater than that after in-the-bag implantation in cataract patients, whereas the decentration of the IOL in CEL patients was significantly greater [21]. The abovementioned findings may be explained by the fact that since our surgical operation required tensioning and fixation of the bilateral sutures, it was difficult to ensure a completely uniform force on both sides with only the surgeon's experience, so it was prone to greater decentration; as most of our patients did not undergo vitrectomy, the more complete vitreous support may explain the lesser tilt.

Zhang F's study showed that IOL tilt and decentration in most patients were distributed on the temporal-inferiorly side [14], and our results showed that the tilt and decentration were mainly nasally inferiorly, which suggests the distribution of IOL positions after trans-scleral sutures was different from that of in-the-bag implantation. In our study, 5.71% (4/70) of CEL patients had an IOL tilt greater than 7°; all four patients had an AL > 26 mm; and the maximum tilt was 9.18° on the nasal side. This patient had an AL > 26 mm and a history of intraoperative anterior vitrectomy. The reason for this patient's large IOL tilt may lie in the fact that the IOL was less supported after the anterior vitrectomy.

We divided the CEL patients into three groups according to the AL, as shown in Table 2 and Figure 3. The postoperative IOL decentration was greater in the CEL patients with an AL ≥ 26 mm than in patients with an AL of less than 26 mm, both on the superior and inferior side. And the IOL tilt was greater in the CEL patients with AL ≥ 26 mm than in patients with AL less than 26 mm on the superior side. This may be explained by the fact that the growth of the eye with a long AL may have stretched the suture of the lens loop fixed to the sclera, thus increasing the chance of postoperative IOL decentration and tilt. As shown in Table 3 and Figure 4, the decentration in superior and inferiority was positively correlated with the AL. This conclusion could be explained by the fact that due to the poorly developed zonular support in CEL patients, the common surgical approach is to fix the IOL in the ciliary sulcus through scleral suture instead of directly into the lens capsule, in the eye with long AL, the IOL may be easily stretched and hence more likely to lead to a large decentration.

There are several limitations in this study. Although the patients came from different regions of China, most were from southern China, which might not represent the whole population with CEL. Although the majority of patients in this study were older than 6 years old, there was still a large age span in our patients and the accuracy of our conclusions may be affected to some extent. More studies are needed in the future to further investigate the relationship between age and IOL position. And this study is a cross-sectional study, and more relevant longitudinal studies are still needed in the future to better understand the distribution and changing trend of tilt and decentration of IOL in CEL patients. However, this study is important as it uses a large sample size focused on the characteristics of the tilt and decentration of the IOL after trans-scleral fixation in CEL patients.

## 5. Conclusion

The results of this study indicate that the trans-scleral sutured posterior-chamber IOL in CEL patients may lead to great decentration. In addition, IOL decentration is positively associated with AL in CEL patients. In future clinical practice, improvements in the surgical approach to CEL patients should be enhanced to reduce the decentration of the IOL. Emphasis should be placed on individualized evaluation and treatment of patients, especially those with long eye axes, to minimize IOL decentration.

## Figures and Tables

**Table 1:** The demographic and clinical features of 70 CEL patients.

Characteristics	Value
Patients/eyes, n	70/70
Age (yrs), mean (SD)	9.77 (5.13)
Male/female, n	33/37
Axial length (mm), mean (SD)	24.86 (1.80)
WTW (mm), mean (SD)	4.67 (1.45)
SE (D)	−1.625
Anterior vitrectomy (n, %)	6 (8.57)

SD = standard deviation.

**Table 2:** IOL position parameters of CEL patients according to axial length.

	Total	AL < 24 mm	24 mm ≤ AL < 26 mm	AL ≥ 26 mm	*P* value
*Tilt (degree)*					

Temporally	2.21 ± 1.53	2.22 ± 1.46	2.38 ± 1.64	2.19 ± 1.58	0.963
Nasally	−1.84 ± 2.04	−1.56 ± 1.52	−1.39 ± 1.27	−1.83 ± 1.66	0.897
Superiorly	2.22 ± 2.18	1.34 ± 1.77	1.89 ± 2.00	3.50 ± 2.71	0.031
Inferiorly	−1.70 ± 1.62	−1.19 ± 1.63	−1.65 ± 1.72	−1.88 ± 1.65	0.523

*Decentration (mm)*					

Temporally	0.49 ± 0.38	0.46 ± 0.25	0.38 ± 0.39	0.52 ± 0.41	0.795
Nasally	−0.69 ± 0.46	−0.53 ± 0.27	−0.74 ± 0.37	−0.56 ± 0.19	0.194
Superiorly	0.72 ± 0.58	0.46 ± 0.25	0.48 ± 0.22	0.72 ± 0.28	0.045
Inferiorly	−0.68 ± 0.54	−0.44 ± 0.26	−0.44 ± 0.46	−0.94 ± 0.56	0.033

**Table 3:** Spearman correlation between clinical features and IOL position.

	Age (yrs)		Sex (female)		WTW (mm)		SE (D)			AL (mm)		ACD (mm)	
	R value	*P* value	R value	*P* value	R value	*P* value	R value	*P* value		R value	*P* value	R value	*P* value
*Tilt*													

Temporally	0.06	0.572	0.33	0.171	0.612	0.312	0.21	0.209		0.02	0.926	0.04	0.462
Nasally	−0.35	0.156	−0.14	0.233	0.526	0.440	0.11	0.621		0.09	0.571	0.10	0.701
Superiorly	0.17	0.725	−0.12	0.501	0.307	0.105	0.10	0.715		0.382	0.041	0.12	0.124
Inferiorly	0.18	0.236	0.09	0.325	0.235	0.303	0.21	0.269		0.07	0.702	0.17	0.506

*Decentration*													

Temporally	0.15	0.314	−0.13	0.163	0.31	0.178	0.14	0.270	−0.08		0.700	0.13	0.423
Nasally	−0.18	0.205	0.23	0.421	−0.27	0.590	0.06	0.19	−0.08		0.635	0.11	0.502
Superiorly	0.23	0.604	0.10	0.121	0.11	0.231	0.12	0.352	0.44		0.019	0.21	0.787
Inferiorly	−0.31	0.215	−0.17	0.304	−0.19	0.331	0.13	0.639	0.542		0.006	−0.31	0.169

## Data Availability

The data used to support the findings of this study are available from the corresponding author upon request.
